# Thermal Properties and Structural Features of Multilayer Films Based on Chitosan and Anionic Polysaccharides

**DOI:** 10.3390/biom11050762

**Published:** 2021-05-19

**Authors:** Galina N. Gubanova, Valentina A. Petrova, Svetlana V. Kononova, Elena N. Popova, Valentina E. Smirnova, Alexander N. Bugrov, Vera V. Klechkovskaya, Yury A. Skorik

**Affiliations:** 1 Institute of Macromolecular Compounds of the Russian Academy of Sciences, Bolshoi pr. VO 31, 199004 St. Petersburg, Russia; gubanovagn@yandex.ru (G.N.G.); valentina_petrova_49@mail.ru (V.A.P.); svetlanavkononova@gmail.com (S.V.K.); popovaen@hq.macro.ru (E.N.P.); ves@hq.macro.ru (V.E.S.); bugrov.an@mail.ru (A.N.B.); 2 Department of Physical Chemistry, Saint Petersburg Electrotechnical University “LETI”, ul. Professora Popova 5, 197376 St. Petersburg, Russia; 3 Federal Scientific Research Centre “Crystallography and Photonics”, Shubnikov Institute of Crystallography, Russian Academy of Sciences, Leninskiy pr. 59, 119333 Moscow, Russia; klechvv@crys.ras.ru

**Keywords:** chitosan, polyelectrolyte complex, sulfoethyl cellulose, alginic acid, hyaluronic acid, thermal analysis, dynamical mechanical analysis

## Abstract

This study investigates the thermal and structural properties of multilayer composites based on chitosan (CS) and polyanions with different functionalities, including sodium sulfoethyl cellulose (SEC), sodium alginate (ALG), and sodium hyaluronate (HA). Unlike polyelectrolyte complexes (PECs) obtained by polymer mixing, the formation of a PEC layer by a process of layer-by-layer deposition of oppositely charged polymers is accompanied by the transformation of the CS polymorphic state, and this affects the relaxation and thermal properties of the resulting multilayer composite. X-ray diffraction analysis showed that the formation of the PEC layer in the CS/SEC multilayer film is accompanied by crystallization of the CS chains and the formation of a predominantly anhydrous CS modification. Thermogravimetric analysis of the CS/SEC film registers a high-temperature peak associated with the thermal decomposition of crystalline CS in the PEC composition. According to the dynamic mechanical analysis, the CS/SEC composite was characterized by a single glass transition temperature, indicating a strong interaction between the layers when using SEC (a strong acid salt) as the counterion to CS. For multilayer composites with weak polyacid salts (ALG and HA), the crystallization of CS in the PEC layer is weaker, as reflected in the thermal degradation of these films. A high-temperature peak is recorded in the thermal decomposition of CS/HA and is absent in the case of CS/ALG. Dynamic mechanical analysis of the CS/ALG composite showed two glass transition temperatures close to those of the original polymers, indicating weak PEC formation. The CS/HA composite showed an intermediate response. Thus, the effect of the PEC layer on the properties of the poly-layer composites decreases in the order CS/SEC > CS/HA > CS/ALG.

## 1. Introduction

Chitosan (CS) possesses unique biological and physicochemical properties that make its polyelectrolyte complexes (PECs) highly useful in medicine, in the pharmaceutical and food industries, and in membrane technologies [1,2,3]. Polyelectrolyte membranes can be manufactured by various techniques (e.g., preparation of a film from the solution containing polyelectrolyte mixture, immersion of a polyelectrolyte film into solution of another polyelectrolyte, etc.). However, the most efficient technique for the preparation of pervaporation membranes is the layer-by-layer deposition of polyelectrolytes [4].

In our previous work, we described a direct method developed to study the structure of thin polymer layers in multilayer composite films, with particular emphasis on the structure of the PEC layer in a CS/sulfoethyl cellulose (SEC) composite [5]. We found that the electrostatic interactions occurring during PEC formation led to appearance of “sites” in the polymer network that initiated crystallization of CS within the PEC layer, as evidenced by the appearance of a reflection in the diffractograms at 2*θ* about 15°. A similar reflection appeared during the hydrothermal treatment of a CS film, and this was interpreted as the transformation of a hydrated polymorphic modification into an anhydrous polymorph [6].

Our previous investigations have explored the properties of multilayer composites based on combinations of CS with various polyanions, such as sulfoethyl cellulose, sodium alginate (ALG), sodium hyaluronate (HA), and carrageenan (CAR), that vary in their functionalities and structures [7]. For all of the studied polymer pairs, the formation of PEC at the phase interface was accompanied to varying degrees by a transformation of the CS structure from an amorphous hydrated form into a crystalline anhydrous polymorph. The degree of transformation of CS into an anhydrous polymorph depends on the steric availability, amount, and basicity/acidity of the oppositely charged ionic groups that form the PEC sites. Scanning electron microscopy and energy-dispersive microanalysis further disclosed the elemental composition and layer thicknesses (including the PEC layer thickness) of multilayer composite membranes [5,7].

Based on the information available in the literature on the structural features of CS [8], we attempted to determine the structure of PECs formed at the interlayer boundary in CS-based multilayer composites [4,5,7,9]. Presumably, the process of CS structure formation in the PEC region affects the microstructure of both adjacent layers. The structure of the formed PEC is related to the strength and rate of the polyelectrolyte interaction. Since the formation of the PEC occurs when the polycationic CS gel film contacts the polyanion solution, a mutual diffusion of polymers occurs at the interlayer boundary. Thus, two processes—the mutual diffusion and the formation of polyelectrolyte contacts—occur simultaneously. In the case of a strong polyelectrolyte interaction (e.g., CS-SEC), mutual diffusion slows down as a result of poly-ion fixing interactions and a well-defined layer interface forms. In the case of weak polyelectrolyte interaction, the mutually diffusing polymers have time to undergo deeper penetration into the layers. As a result, a blurred interface is formed, as described in detail in [7]. Neither just how completely the polyelectrolyte contacts at the interface are realized in each case nor whether we know the strength of the contacts that are formed or how this correlates with the microstructure of the sample are clear. For this reason, in this work, we attempted to involve thermophysical methods in solving these questions.

A number of studies [10,11,12,13] have applied thermogravimetric analysis (TGA) and differential scanning calorimetry (DSC) to confirm the formation of PEC between CS and different polysaccharides (e.g., pectin, carboxymethyl cellulose, ALG, and HA). The most informative method for determination of the water state in polysaccharides and PECs is DSC. As shown previously [14,15], the degree of hydration of polysaccharides depends on the molecular and supramolecular structures of the polymers. The plasticizing action of water and the high hygroscopicity of CS hinder the determination of the glass transition temperature, thereby causing a considerable scatter of the experimental data [16,17]. 

One common method for determining the temperature of relaxation transitions in polymers is dynamic mechanical analysis (DMA), which has a greater sensitivity for molecular motion compared with other methods of thermal analysis [18]. The DMA method has been used for the analysis of relaxation transitions in branched copolymers based on CS and dextran, as reported in [19]. In particular, that paper contains a detailed discussion of the contradictions in the literature related to the determination of the CS glass transition temperature. The thermophysical properties of CS in the basic and salt forms were also studied by DMA in [20]. The high glass transition temperatures obtained for CS confirm the conclusions made in [19].

An ALG-CS complex with various degrees of poly-ion interactions was studied by both TGA and DMA in [21]. The thermal properties and thermal stability of HA, CS, and PEC films were investigated using TGA in [11]. The thermal decomposition of the PEC film occurred at ≈280 °C, and the TGA results showed a rapid increase in the thermal decomposition temperature of PEC films as the CS content increased. The PEC films in that paper were prepared by dissolving the two components at various ratios in an aqueous 50 wt.% formic acid solution.

The thermal properties of the CS/HA complex were studied in [22]. The PECs of chitosan and HA were prepared by mixing various weight ratios of two oppositely charged polymers at various pH values. DSC was used for quantitative determination of the amounts of freezing and non-freezing water. DSC, TGA, and XRD have been used to characterize CS/ALG polyelectrolyte complexes [23] prepared by freeze-drying a precipitate from sufficient mixtures of the two polymers. After the complex formation, the crystalline structure of CS disappeared, and the PECs showed an amorphous morphology. In [24], PEC membranes made by blending 84% deacetylated CS and ALG were investigated for use in a direct methanol fuel cell. XRD was used to observe the effects of blending on the crystallinity of the membranes. The thermal stability of the membranes was determined by thermal analysis.

Our examination of a series of multilayer CS-based composites revealed an effect of the structure and properties of the PEC layer on the properties of membranes [4,9,25,26]. A two-layer CS_b_-PEC_HA_ film was obtained after the conversion of CS from its salt form (CS_s_) to the basic form (CS_b_) and after removal of the HA layer. The thermal decomposition of the film was characterized by three peaks and a maximum weight loss rate at 220, 293, and 360 °C [9]. The high-temperature peak at 360 °C was considered to reflect the degradation of the PEC_HA_ layer containing anhydrous CS. Two low-temperature peaks were attributed to the thermal decomposition of two CS polymorphs, as confirmed by X-ray diffraction. 

Thus, thermal analysis approaches are widely used to study the properties of PECs based on natural and synthetic polymers. However, thermal analysis has not been used to study the PEC layer in multilayer composite films and membranes. The aims of the present work were to study the thermophysical properties of multilayer composite films based on CS and anionic polysaccharides (specifically SEC, ALG, and HA) and to investigate the effects of structurally different polyanions on the relaxation and the structural characteristics of multilayer CS/polyanion composites. The fundamental difference and complexity of the present research is the determination of the thermal characteristics of the structural elements formed during the formation of a multilayer composite containing a PEC layer in its composition. From this point of view, all of the studied objects are investigated for the first time and the information obtained is of great importance for understanding the process of multilayer PEC composite formation and its properties, including functional properties.

## 2. Materials and Methods

### 2.1. Materials

This study was performed using crab shell chitosan (Bioprogress, Shchelkovo, Russia) with a viscosity average molecular weight (M_η_) of 1.6 × 10^5^ and a degree of deacetylation of 0.80 [9], sodium hyaluronate (Shandong Focuchem Biotech Co., Qufu, Shandong, China) with M_η_ of 5.4 × 10^4^ [27], sodium alginate (Qingdao Bright Moon Seaweed Group Co. Ltd., Qingdao, Shandong, China) with M_η_ of 1.3 × 10^5^ [28], and sodium sulfoethyl cellulose with M_η_ of 1.4 × 10^4^ and a degree of substitution of 0.80 (the sample was prepared and characterized according to [4]).

### 2.2. Preparation of Composite Films 

Composite films were obtained using the layer-by-layer deposition of polymer solutions. A 2% CS solution in 2% acetic acid was deposited from a spinneret onto a balanced glass substrate and dried to the gel-like state. A 2% aqueous solution of polyanion was then deposited onto the surface of the layer, and the films were dried at room temperature. The resulting complex film (25–30 μm thick) can be considered a three-layer system containing chitosan acetate (salt form, CS_s_), the polyanion, and the PEC layer formed between the two polyelectrolyte layers. Treatment of this film with 2% ammonia in ethanol transformed the CS_s_ into the water-insoluble basic form (CS_b_), thereby allowing the removal of the water-soluble polyanion layer by extraction with water for analysis of the structure of the PEC layer. Various polyanions were used to prepare three-layer films (CS_s_–PEC_SEC_–SEC, CS_s_–PEC_HA_–HA, and CS_s_–PEC_ALG_–ALG) and two-layer films (CS_b_–PEC_SEC_, CS_b_–PEC_HA_, and CS_b_–PEC_ALG_).

The structure of the composite films was compared by preparing a film containing an anhydrous polymorph of CS. For this, the CS_s_ film was processed by hydrothermal treatment. In this case, the CS_s_ film, weighing 0.23 g, was placed into a 15 mL Teflon cell, which was 2/3 filled with distilled water. After that, the hermetically sealed steel autoclave was heated in an oven to 200 °C. The sample was held under isothermal conditions for 20 min at 100 atm. The pressure inside the autoclave for the selected temperature was set in accordance with the cell filling factor based on the Kennedy table [29]. At the end of the temperature treatment, the autoclave was cooled down, together with the oven, to room temperature. The film was removed from the cell and dried in air at 60 °C to constant weight.

### 2.3. Methods

TGA was performed using a TG 209 F1 (Erich NETZSCH GmbH & Co, Selb, Germany) setup in a temperature range from 30 to 600 °C, again at a 10 °C·min^−1^ heating rate, in argon medium. The sample mass was 2–3 mg. 

DSC analysis of the samples was carried out using a DSC 204 F1 (Erich NETZSCH GmbH & Co, Selb, Germany) heat flow differential scanning calorimeter in a temperature range from 30 to 250 °C in an argon atmosphere; the heating rate was 10 °C·min^−1^.

A DMA 242 C setup (Erich NETZSCH GmbH & Co, Selb, Germany) was used to measure the temperature dependence of the dynamic mechanical characteristics of the films during stretching (Young’s modulus *E’*, loss modulus *E”*, and mechanical loss tangent *tgδ*). The measurements were carried out at a frequency of 1 Hz and a heating rate of 5 °C·min^−1^. The temperatures of the relaxation transitions were determined from the positions of the maxima or from inflexions of the curves for dynamic parameters.

The X-ray diffraction analysis was performed using a Miniflex 600 (Rigaku Technologies, Inc., Tokyo, Japan) diffractometer (U = 40 kV, J = 15 mA, and a range of angles 2*θ* from 5 to 50).

Cross-sectional images of the films were taken on a Scios scanning electron microscope (Field Electron and Ion Company, Hillsboro, OR, USA) with a FEG electron source.

## 3. Results and Discussion

### 3.1. Thermal Analysis of the CS Polyanion Films

Multilayer films were studied by thermal analysis (TGA and DSC), with the assumption that each layer has its own individual thermal stability parameters.

**Figure 1:**
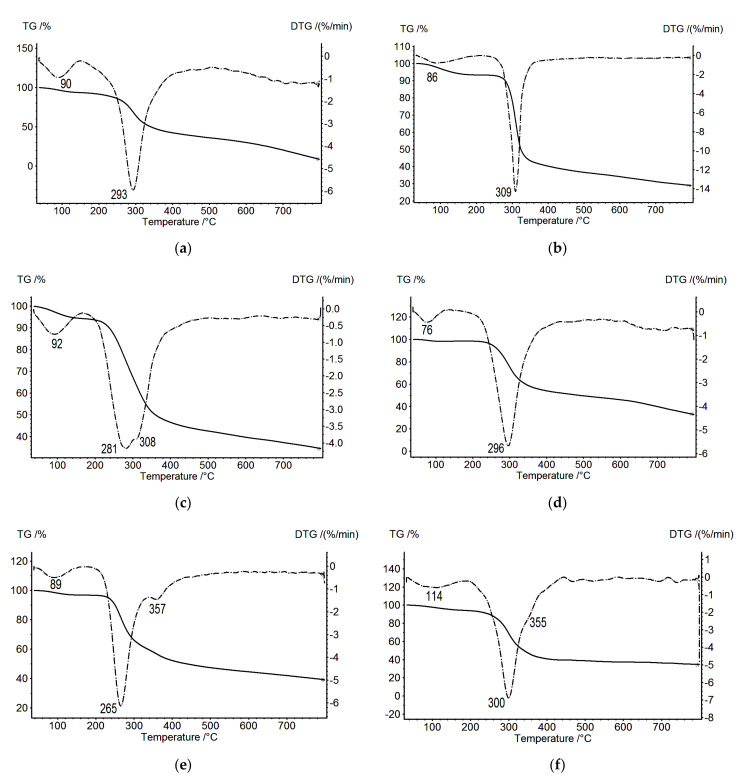
Thermogravimetric (TG; solid lines) and differential thermogravimetric (DTG; dotted lines) curves: (**a**) CS_s_, (**b**) SEC, (**c**) CS_s_–PEC_SEC_–SEC, (**d**) CS_b_, (**e**) CS_b_–PEC_SEC_, and (**f**) CS_b_ after hydrothermal treatment.

Figure 1 presents the TGA data (TG, solid line) and differential thermogravimetric analyses (DTG, dotted line) of CS in the salt form (CS_s_), SEC, and a three-layer film (CS_s_–PEC_SEC_–SEC). Each thermal decomposition process for CS_s_ (Figure 1a, DTG curve) and SEC (Figure 1b, DTG curve) consists of one stage, with the maximum mass loss rates observed at 293 and 309 °C, respectively. Thermal degradation of the CS_s_–PEC_SEC_–SEC composite (Figure 1c) is a more complex process; the differential curve of the mass loss is bimodal and contains two maxima (at 281 and at 308 °C), where the last maximum coincides with the temperature of the maximum decomposition rate of SEC (Figure 1b). The temperature maximum at 281 °C differs from the temperature of the maximum decomposition rate of CS_s_ (Figure 1a), thereby allowing its attribution to the total decomposition of the PEC layer and CS_s_. This is confirmed by a noticeable broadening of the DTG curve of the CS_s_–PEC_SEC_–SEC composite when compared to the curves of the original polymers.

The thermal analysis of two-layer films containing CS_b_ and PEC layers was particularly informative. The two-layer CS_b_–PEC_SEC_ film demonstrates two decomposition peaks located at 265 and 357 °C (Figure 1e); the first peak does not coincide with the decomposition maxima of CS_b_ (Figure 1d), whereas the second peak (at 357 °C) was not observed on the TG and DTG curves presented in Figure 1a–c.

Figure 1f shows the TGA curve for a CS_s_ film that had been subjected to hydrothermal treatment at 200 °C for 20 min. Hydrothermal treatment is known to transform a hydrated CS_s_ polymorph into an anhydrous crystalline polymorph [6]. This transformation is accompanied by the appearance of intense reflections in the X-ray diffractograms (2*θ* = 15, 21 and 22.4°). We observed a similar pattern in our previous work [5] with a CS_s_ sample subjected to hydrothermal treatment. At the same time, a weak reflection at 2*θ* = 10° was also registered, indicating the presence of hydrated CS in the sample [5]. According to [6], the presence of this reflection and its absence in the diffractogram obtained after hydrothermal treatment of the film can be explained by the presence of water molecules between the CS polymer chains; these molecules are arranged along the (010) crystallographic plane. Since the thermal decomposition curve of this sample (Figure 1f) is bimodal, we assumed, in our case, that two CS polymorphs (hydrated and anhydrous) are present in the sample. Indeed, the decomposition peak at 300 °C is very close to the decomposition peak of CS_b_ (Figure 1d). Therefore, the high-temperature maximum at 355 °C can be attributed to anhydrous CS. Completely anhydrous CS can be obtained after the sample was subjected to orientation tension in water at 95 °C before hydrothermal treatment [30].

A bimodal decomposition curve was also observed for the CS_b_–PEC_SEC_ sample (Figure 1e). The weakly pronounced high-temperature peak at 357 °C can be attributed to the anhydrous CS polymorph. The presence of this modification is confirmed by the appearance of the signal at 2*θ* = 15° in the diffractograms [6]. The first peak at 265 °C may be connected with the decomposition of the hydrated CS polymorph. Conformational variants for CS are not resolved for most polymorphs. For one of the hydrated forms of CS, the crystal structure was determined based on X-ray diffraction data [31].

**Figure 2:**
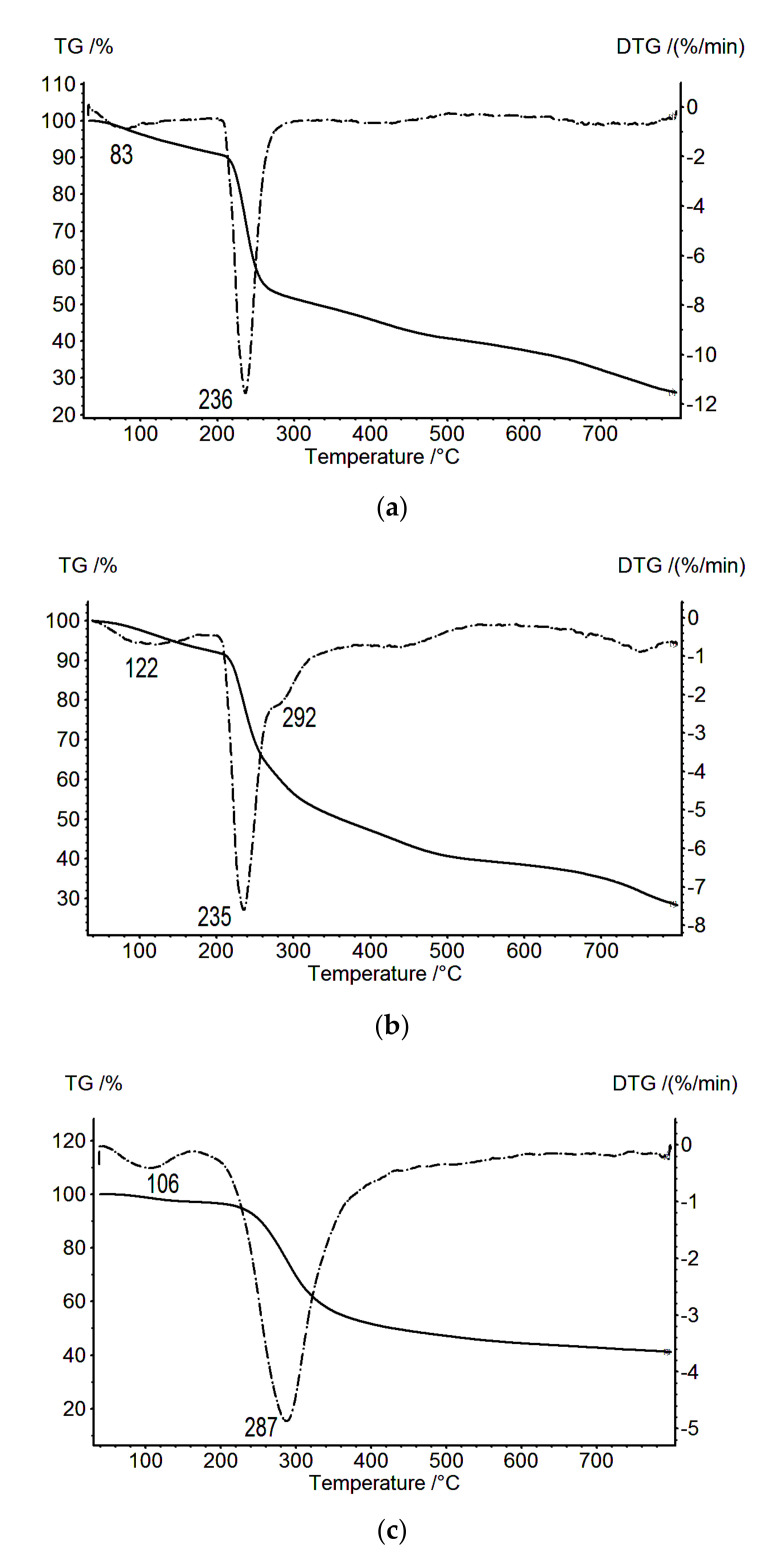
Thermogravimetric (TG; solid lines) and differential thermogravimetric (DTG; dotted lines) curves: (**a**) ALG, (**b**) CS_s_–PEC_ALG_–ALG, and (**c**) CS_b_–PEC_ALG_.

Figure 2 presents the TG and DTG data obtained for the ALG film and the composite CS_s_–PEC_ALG_–ALG and CS_b_–PEC_ALG_ films. The thermo-destruction of the CS_s_–PEC_ALG_–ALG composites occurs in two stages, with the maximum mass loss rates observed at 235.0 and 292 °C (Figure 2b). The first temperature essentially coincides with the temperature of the destruction of the ALG polyanion (Figure 2a), while the second maximum is close to the degradation temperature of CS. The second observation is explained by the presence of a loose network of polyelectrolyte contacts (ionic crosslinks), and it may indicate a low stability of the obtained PEC_ALG_. A significant difference is also noted in the thermal behavior of the multilayer composites in the case of formation of a weak PEC.

In the case of the CS_b_–PEC_ALG_ composite layer (Figure 2c), only one destruction temperature was registered in the DTG experiments (287 °C), and this temperature differed from the degradation temperatures of the individual components of this complex. Presumably, this corresponds to the degradation of one of the CS polymorphs. No degradation is registered for the PEC incorporated into the CS_b_–PEC_ALG_ two-layer film, indicating an apparent overlap of the CS and PEC thermo-destruction processes in the CS_b_–PEC_ALG_ two-layer film (as indicated by the large half-width of its DTG peak, Figure 2c). The weak CS structurization in the CS_b_–PEC_ALG_ composite is related to the ALG structure, which leads to the formation of a diffuse PEC layer [7].

**Figure 3:**
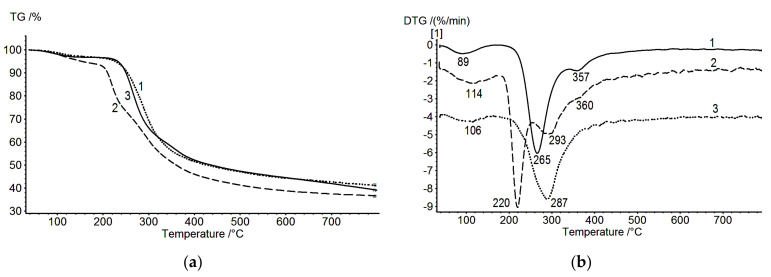
TG (**a**) and DTG (**b**) curves: CS_b_–PEC_SEC_ (1), CS_b_–PEC_HA_ (2) [9], and CS_b_–PEC_ALG_ (3).

The CS/HA composite occupies an intermediate position relative to the other two composites [9]. Figure 3a shows the TGA data for two-layer samples CS_b_-PEC_SEC_ (solid line), CS_b_–PEC_ALG_ (dash-dotted line), and CS_b_–PEC_HA_ (dotted line). The weight loss is significantly higher for the CS_b_–PEC_HA_ two-layer film than for the other composites. A comparison of the effect of the type of polyanion on the structure of the PEC layer is depicted in Figure 3b, which shows the results for the thermal decomposition of two-layer films obtained using polyanions of different functionality and structure.

The thermal degradation of the CS_b_–PEC_HA_ composite film proceeds in three stages (Figure 3b), with a maximum weight loss rate at 220, 293, and 360 °C. The presence of at least two polymorphic modifications of CS (hydrated and anhydrous) in the CS_b_–PEC_HA_ film is confirmed by the X-ray diffraction as evidenced by the signal at 2*θ* = 10°, typical for the hydrated CS, and at 2*θ* = 15°, typical for the anhydrous CS polymorph [9]. The peaks at 220 and 293 °C can be attributed to the degradation of the hydrated CS polymorph, while the peak at 360 °C is associated with the degradation of the PEC_HA_ containing the anhydrous polymorph of CS in its structure. Unlike the CS/SEC polymer pair, the CS/HA pair shows a more intense signal for the hydrated CS than for the anhydrous CS. Therefore, the ability of HA to retain water influences the chemical composition of the film in the region of the PEC. Consequently, the PEC layer for this pair is more hydrophilic, as indicated by the higher swelling degree of the PEC_HA_ isolated from the three-layer composite (compared to that of PEC_SEC_ [7]). However, the formation of hydrated CS in this region is only indirectly related to this property of HA, which, in a way, is a supplier of construction materials (water molecules) for the CS–HA interphase boundary. At the same time, as demonstrated experimentally, the formation of the CS structure in a near-boundary area proceeds during contact between CS film (in the salt form CS_s_) and the aqueous solution of HA, and this formation is followed by the removal of water (drying in air).

In this case, at least two processes occur: one is an interaction between the oppositely charged groups (ionic crosslinking), and the other is diffusion of the polyanion (HA) into the region of the CS_s_ gel film. Each of these processes is accompanied by changes in the “instantaneous” polysaccharide conformation, and each influences the polymer conformational features in the resulting composite. Since HA is a weak acid (i.e., it contains carboxyl groups), the rate of the exchange reaction involved in the formation of PEC is comparable to the rate of polysaccharide diffusion into the interphase region (as observed during the formation of certain simplex membranes [9]). These processes then lead to the formation of a multilayer film containing polymer layers of various microstructures with a more diffuse interphase boundary than is found in the CS–SEC film [4,7].

The results discussed in the present work demonstrate that a conformational rearrangement in the PEC area of the CS/HA composite leads to the formation of two polymorphs, with the hydrated form predominating. The high-temperature peak observed during thermal degradation of CS/HA, which corresponds to the anhydrous form, is less pronounced than in the case of CS/SEC (Figure 3b, curves 1 and 2). When CS interacts with a strong polyacid anion (SEC), the rate of the exchange reaction yielding PEC_SEC_ is very rapid, whereas the diffusion of polysaccharides at the interphase boundary is insignificant [4]. In this case, only the anhydrous CS polymorph is formed in the PEC_SEC_ region, as demonstrated above. The most interesting fact is that conformations of the polymorphs formed in PEC_HA_ and PEC_SEC_ differ from the conformations of CS_b_ and the initial CS_s_, as seen from the above data (Figure 1 and Figure 3). These conclusions agree well with recent reports on two-layer film structures [4,7,9].

The polyanions that lead to the formation of these two groups of PECs differ in the nature of the ionic groups in their structure. In the first case, the ionic groups are strong acid salts (R–SO_3_– for SEC), whereas, in the second case, they are weak acid salts (carboxylate groups for HA and ALG). The formation of a PEC between CS and a strong acid salt proceeds rapidly, whereas in the second case, the process is relatively slow. This slow complex formation is accompanied by interpenetration of the polymers, so that the interface between the formed layers becomes diffuse. Presumably, the weak dissociation of a polyacid leads to the coexistence of two forms (acidic and ionic/salt forms) in the resulting film.

No PEC degradation is registered for the CS_b_–PEC_ALG_ two-layer film (Figure 3b, curve 3). The low degree of CS structuring in the CS_b_–PEC_ALG_ composite is associated with the irregular ALG structure (block copolymer of (1→4) linked β-D-mannuronate and α-L-guluronate), which leads to a lesser degree of ionic cross-linking.

All of the TGA and DTG curves depicted in Figure 3 indicate a weight loss before the onset of thermal destruction; this loss is associated with the release of water from the two-layer CS-based composites examined here. The smallest maximum in the rate of weight loss in this region (89 °C) is observed for the CS_b_–PEC_SEC_ two-layer film. The completion of the water release for this sample is recorded at 120 °C. As shown previously [10,14], water bound by hydrogen bonds with polysaccharides is released in this region. The release of water associated with carboxyl groups is shifted toward higher temperatures (above 160 °C). In our case, the carboxyl groups contain two poly-ions: HA and ALG. Consequently, the shift in the maxima of the rates of release of bound water observed on the DTG curves (Figure 3b, curves 2 and 3) is quite expected. In this case, the release of bound water is recorded until the beginning of thermal decomposition. The amount of released bound water was estimated by the DSC method and amounted to 211.5, 457.5, and 320.5 J/g for CS_b_-PEC_SEC_, CS_b_-PEC_HA_, and CS_b_-PEC_ALG_, respectively.

### 3.2. Scanning Electron Microscopy

**Figure 4:**
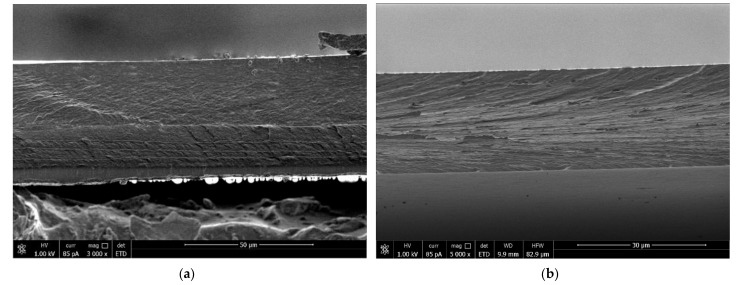
SEM images of cross sections of CS_s_–PEC_SEC_–SEC (**a**) and CS_s_–PEC_HA_–HA (**b**).

Comparative analysis of SEM images of cross sections of multilayer composites revealed clear contouring of the PEC layers in CS/SEC composites (Figure 4a). In other cases (e.g., CS/HA sample), a boundary emerged between the CS and the polyanion, but the PEC layer was poorly visualized (Figure 4b). The PEC layer in the CS_s_–PEC_SEC_–SEC film is dense, while that in the CS_s_–PEC_HA_–HA film is more loose, fuzzy, and contains defects. These data are in a good agreement with TGA data discussed above.

### 3.3. X-ray Diffraction Analysis of the CS Polyanion Films

**Figure 5:**
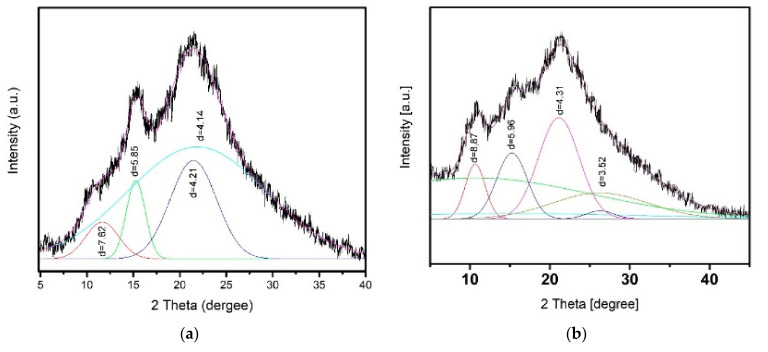
X-ray diffractograms of two-layer films (after removal of the water-soluble polyanion layer): (**a**) CS_b_–PEC_SEC_; (**b**) CS_b_–PEC_HA_, obtained from the PEC side.

Unlike the X-ray diffraction patterns presented in [7,9], a more detailed X-ray diffraction analysis was performed for the two-layer CS_b_–PEC_SEC_ and CS_b_–PEC_HA_ samples (Figure 5). This analysis was not carried out for the CS_b_–PEC_ALG_ sample because of its diffuse PEC layer and the weak signal in the region 2*θ*: ≈15°.

Under certain conditions, many polymers are known to crystallize and form crystal lattices throughout the whole volume of a sample (or in a portion of a sample). The diffraction maxima in X-ray patterns were defined by conducting a profile analysis (plotting Gaussian functions [32], which takes into account the various types of peak widening (including instrumental distortion, etc.), thereby allowing refinement of the peak positions. Analysis of the X-ray diffractograms demonstrated that the formation of the CS_b_–PEC_SEC_ composite is accompanied by crystallization of the CS chains, with a prevailing appearance of the anhydrous CS polymorph. The X-ray patterns contain an intense reflection at 2*θ* ≈ 15° (the lattice spacing *d* = 5.85 Å); this reflection is typical of an anhydrous polymorph. In addition, a weak reflection at 2*θ*, ≈10° (*d* = 7.62 Å), occurs that is attributed to hydrated CS. 

By contrast, the X-ray diffractograms of the CS_b_–PEC_HA_ are quite different. The reflections at 2*θ* ≈ 15° (*d* = 5.96 Å) and ≈10° (*d* = 8.87 Å) have similar intensities, indicating the presence of equal proportions of hydrated and anhydrous CS modifications in the film. One explanation for the differences in the interplanar distances in the hydrated phase of these two samples may be that the polymer molecules in a film are not completely packed into a crystal lattice; rather, the crystalline areas are interspersed among amorphous regions, thereby creating various defects in the polymer packing. These differences are related to specific features of the interaction of SEC and HA chains with CS (molecular structure, degree of substitution by polar groups, etc.). The relative degrees of crystallinity for these samples were estimated according to the intensity conservation law, which holds that intensity is determined by the volume integral of the electron density squared of an object [32]. The following degrees of crystallinity were found: 35.3% for CS_b_–PEC_SEC_ and 27.0% for CS_b_–PEC_HA_. A noticeable difference was observed in the thermal behavior of the multilayer composites when a weak PEC was formed.

### 3.4. Dynamic Mechanical Analysis of the CS Polyanion Films

The influence of the PEC layer (and, therefore, the factors determining its structural and morphological features) should manifest in the relaxation properties of the composites, particularly in the glass transition temperatures. The temperatures of the relaxation transitions were estimated from the temperatures of the maxima or inflexions on the curves *tgδ*, which describe the dynamic parameters of the samples.

**Figure 6:**
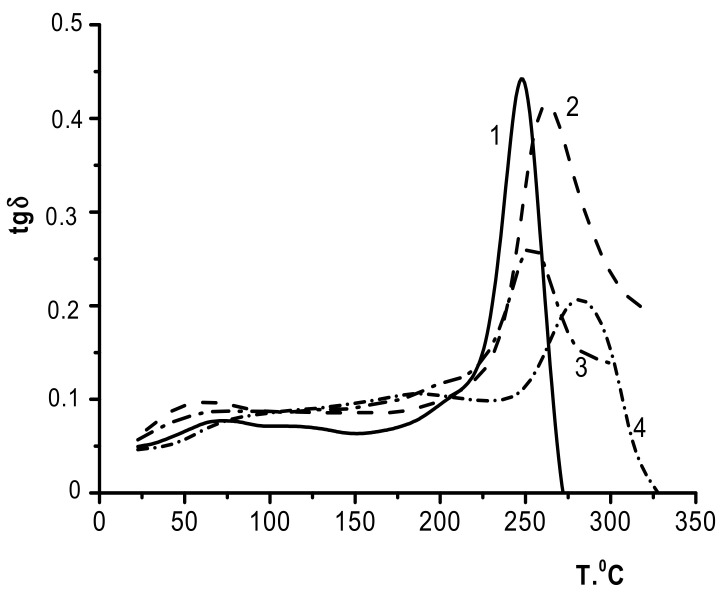
Temperature dependences of the mechanical loss tangent: 1, SEC; 2, CS_b_–PEC_SEC_; 3, CS_s_–PEC_SEC_–SEC; and 4, CS_b_.

During the formation of a strong PEC between CS and SEC, the two-layer and three-layer composites are characterized by only a single glass transition temperature (Figure 6, curves 3 and 2, respectively). As can be seen, no noticeable transitions were observed up to 200 °C for all samples. The week maxima of *tgδ* in the 150–200° region (Figure 6, curve 4) is related to molecular mobility of chitosan caused by the removal of absorbed and residues of acetic acid [20].

The maxima for the *tg*δ vs. *T* curves correspond to the glass transition temperatures of the samples (254 °C for CS–PEC_SEC_–SEC and 262 °C for CS_b_–PEC_SEC_); these are lower than the glass transition temperature for CS_b_ (265 °C) but higher than the glass transition temperature for SEC (248 °C). Therefore, upon formation of a CS/SEC polyelectrolyte complex, each two-layer and three-layer composite demonstrates a single glass transition temperature (Figure 6, curves 3 and 2, respectively) and can be considered an integrated system that represents a combination of components at the molecular level.

We made several attempts to subject the three-layer CS_s_–PEC_HA_–HA films to DMA; however, these attempts were not successful. The samples broke at 210–215 °C (which approximately corresponded to the HA glass transition temperature). Similarly, the two-layer CS_b_–PEC_HA_ samples were successfully tested only at temperatures not exceeding 350 °C, indicating that the deformation abilities of the component layers are virtually the same.

**Figure 7:**
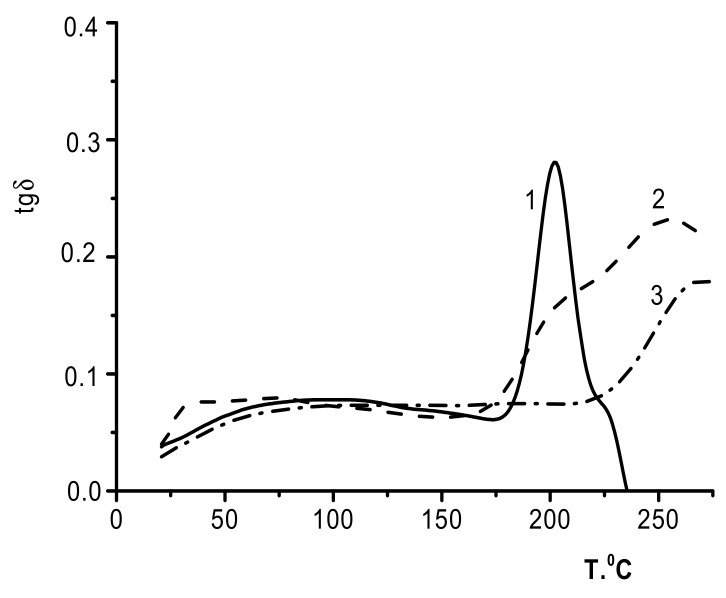
Temperature dependence of the mechanical loss tangent: 1, HA; 2, CS_b_–PEC_HA_; and 3, CS_b_.

Figure 7 presents the temperature dependence of *tg*δ for the HA film (curve 1), the two-layer CS–PEC_HA_ film (curve 2), and the CS_b_ film (curve 3). The *tg*δ curve for the two-layer film has a maximum at 255 °C, which does not coincide with any of the glass transition temperatures of the individual components (i.e., CS_b_ and HA, at 265 and 203 °C, respectively). This curve also contains a shoulder in the low-temperature region that corresponds to the HA glass transition point. This transition can be explained by the presence of a HA phase that develops after the formation of PEC due to the mutual diffusion of the polymer counterions [9].

**Figure 8:**
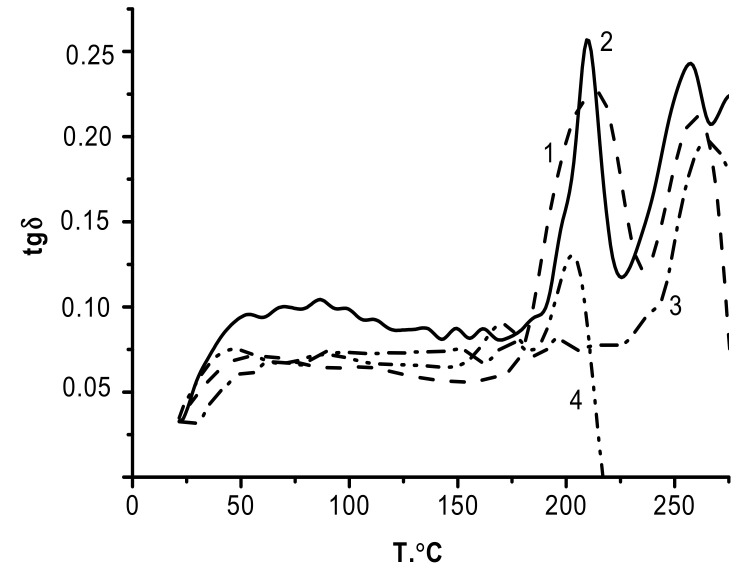
Temperature dependence of the mechanical loss tangent: 1, CS_s_–PEC_ALG_–ALG; 2, CS_b_–PEC_ALG_; 3, CS_b_; and 4, ALG.

The temperature dependence of *tg*δ obtained for CS_s_–PEC_ALG_–ALG (Figure 8, curve 1) contains two maxima (214 °C and 260 °C); these two glass transition temperatures differ only slightly from those of the film components (ALG and CS, curves 4 and 3, respectively).

Therefore, the ALG and CS layers in the CS_s_–PEC_ALG_–ALG composite retain their intrinsic glass transition temperatures, and the formation of the interlayer PEC_ALG_ complex does not influence the relaxation behavior of the neighboring layers. Note that the *tg*δ dependence for CS_b_–PEC_ALG_ has a similar bimodal pattern. In this case, the peaks corresponding to the two glass transition temperatures have significantly lower half-widths and are shifted toward higher temperatures. One unexplained feature is why the DMA curves of the two-layer CS_b_–PEC_ALG_ sample contain a peak with a glass transition temperature close to that of the polyanion, even after the removal of the ALG layer. This is likely due to the slower PEC formation in the case of layer-by-layer deposition of ALG on the CS gel-film.

At the same time, an effect of mutual diffusion prevails in the ionic interactions, which then allows for recording the main α-relaxation transition (T_g_) of ALG.

The polyacids described here can be conventionally divided into two groups according to their abilities to dissociate (i.e., weak and strong polyacids). The rate of formation of a PEC is slower between CS and a weak polyacid anion (HA or ALG) than it is between CS and a strong polyacid anion (SEC). The complex forms rather gradually, so the conformational rearrangements have sufficient time to occur in the swollen polymer. The differences in the trends of the temperature dependences of the mechanical loss tangents for HA and ALG can be assumed to arise due to the different conformational mobilities of HA and ALG. In this connection, our future work will involve in-depth computer modeling studies of the structure of the composites near the polyanion/CS interphase boundary.

## 4. Conclusions

The results obtained in this work suggest that the structure of the PEC layer in a multilayer polymer composite is the feature that controls the relaxation and thermal properties of that composite. The influence of a PEC on the properties of different CS-based composites decreases in the order CS/SEC > CS/HA > CS/ALG, and this effect is more pronounced in two-layer films. Our conclusions agree with the reported data on the influence of the PEC layer on the structural organization and morphology of various composites.

The experimental curves for the thermal degradation of the CS/SEC composite were bimodal, indicating the presence of two CS polymorphs (i.e., hydrated and anhydrous). The two-layer and three-layer composites containing the same polyelectrolyte pair show single glass transition temperatures, indicating that they can be considered integrated systems. The properties of these composites arise due to the presence of strong acid anions (–SO_3_^−^) in the SEC and the complementarity of the SEC structure with that of CS.

Structural studies of two-layer CS/HA and CS/SEC films confirmed the difference in the structural organization of the PEC layers. The formation of the CS/SEC composite is accompanied by crystallization of the CS chains and the formation of a predominantly anhydrous CS polymorph. For the CS/HA composite, the hydrated and anhydrous CS polymorph are present in comparable proportions.

The thermal degradation behavior of the CS/HA sample shows that, in the PEC region, CS undergoes a conformational rearrangement to form two polymorphs with predominant hydrated modifications. The observed glass transition temperature of the two-layer film does not coincide with that of either the CS_b_ or the HA glass transition values, although the curve contains a shoulder in the low-temperature region that is close to the HA glass transition temperature. The difference in the properties of this composite from those of the CS_b_–PEC_SEC_ is associated with the presence of weak acid salts (–COO^-^) in HA as well as the ability of HA to retain water, which, in turn, affects the chemical composition of the composite in the PEC region.

Regarding the CS/ALG polymer pair, the two-layer film demonstrates only one destruction temperature, and this differs from the destruction temperature of either of the individual components. For this polymer pair, we observed two glass transition temperatures that were close to the corresponding parameters of the initial polymers. Thus, the differences in the thermophysical and relaxation properties of the CS_b_–PEC_HA_ and CS_b_–PEC_ALG_ composites containing polyanions with carboxylate groups are associated with differences in the conformational mobility of the polyanions that form them.

Studies on the relaxation and thermal properties of multilayer composite films based on differently charged polysaccharides can provide valuable information for predicting the physicochemical properties of pervaporation membranes and multilayer composite materials for use in tissue engineering and regenerative medicine.

## Data Availability

The data are contained within the article.
